# Probing Cellular Mechanoadaptation Using Cell-Substrate De-Adhesion Dynamics: Experiments and Model

**DOI:** 10.1371/journal.pone.0106915

**Published:** 2014-09-08

**Authors:** Soumya S S, Lakshmi Kavitha Sthanam, Ranjith Padinhateeri, Mandar M. Inamdar, Shamik Sen

**Affiliations:** 1 Department of Civil Engineering, Indian Institute of Technology Bombay, Mumbai, Maharashtra, India; 2 Department of Biosciences and Bioengineering, Indian Institute of Technology Bombay, Mumbai, Maharashtra, India; University of California, San Diego, United States of America

## Abstract

Physical properties of the extracellular matrix (ECM) are known to regulate cellular processes ranging from spreading to differentiation, with alterations in cell phenotype closely associated with changes in physical properties of cells themselves. When plated on substrates of varying stiffness, fibroblasts have been shown to exhibit stiffness matching property, wherein cell cortical stiffness increases in proportion to substrate stiffness up to 5 kPa, and subsequently saturates. Similar mechanoadaptation responses have also been observed in other cell types. Trypsin de-adhesion represents a simple experimental framework for probing the contractile mechanics of adherent cells, with de-adhesion timescales shown to scale inversely with cortical stiffness values. In this study, we combine experiments and computation in deciphering the influence of substrate properties in regulating de-adhesion dynamics of adherent cells. We first show that NIH 3T3 fibroblasts cultured on collagen-coated polyacrylamide hydrogels de-adhere faster on stiffer substrates. Using a simple computational model, we qualitatively show how substrate stiffness and cell-substrate bond breakage rate collectively influence de-adhesion timescales, and also obtain analytical expressions of de-adhesion timescales in certain regimes of the parameter space. Finally, by comparing stiffness-dependent experimental and computational de-adhesion responses, we show that faster de-adhesion on stiffer substrates arises due to force-dependent breakage of cell-matrix adhesions. In addition to illustrating the utility of employing trypsin de-adhesion as a biophysical tool for probing mechanoadaptation, our computational results highlight the collective interplay of substrate properties and bond breakage rate in setting de-adhesion timescales.

## Introduction

The extracellular matrix (ECM) which serves as a scaffold for maintaining the integrity of various tissues, is known to encode a diverse range of physical cues, including stiffness, topography, geometry, ligand spacing and dimensionality [1]. Of these, ECM stiffness has emerged as an important factor and has been shown to modulate a range of cellular processes including spreading [2], motility [3], differentiation [4] and cancer invasion [5]. Such responses to ECM features require a close coupling between cell-matrix adhesions (also called focal adhesions) and the contractile acto-myosin cytoskeleton, leading to active reorganisation of the cytoskeleton and the adhesions [6], [7]. Changes in cellular processes are closely tied to changes in physical properties of the cells, as evidenced by changes in cell cortical stiffness and traction forces exerted by a range of different cell types across substrates of varying stiffness [8].

Trypsin de-adhesion represents a simple method for probing the biophysical properties of adherent cells [9], [10]. In this assay, upon incubation with warmed trypsin, cells round up driven by rapid severing of cell-matrix adhesions. The retraction process obeys sigmoidal kinetics with time constants that track cortical stiffness values. Further, de-adhesion time constants are sensitive to cellular contractility, with contractile activation leading to faster de-adhesion and contractile inhibition leading to delayed de-adhesion. Consequently, the de-adhesion assay has been used for studying modulation of cellular contractility by various features of the ECM. In breast cancer cells, increase in ECM density has been shown to increase protease-mediated ECM degradation in a contractility-dependent manner [11], [12]. In addition to tracking cell rounding during de-adhesion, the pattern of cell movement during de-adhesion, i.e., translation and/or rotation, may differ from cell to cell, and was shown to depend on the spatial anisotropy in cell contractility, bond distribution and bond strength. Specifically, while asymmetry in bond strength and/or bond distribution was shown to cause cell translation, cell rotation required spatial asymmetry in both bond distribution and contractility [13]. These results may help us in understanding the collective influence of contractility, bond distribution and bond strength in modulating random versus persistent cell motility. Taken together, these results illustrate the effectiveness of the trypsin de-adhesion assay for probing cell mechanics.

Significant insight into the contributions of adhesion and contractility to various cellular processes has been obtained via a diverse range of theoretical and computational studies [14]–[16]. Several of these studies have tried to understand the role of substrate properties on cellular responses including spreading and motility [17]–[20]. Some of these studies include traction force localisation in cells [21], the role of substrate stiffness on stress fiber alignment [22], and the role of substrate thickness, stiffness and geometry of adhesion patches on traction forces generated by adherent cells [23], [24]. Though a cell is a highly heterogeneous and dynamic entity, it is not uncommon in the literature to have a simple, linear, isotropic, elastic/visco-elastic description of the cell [25]–[28]. Using a similar description, Mofrad and co-workers demonstrated the viscoelastic behavior of NIH 3T3 cells during cell detachment [29], [30]. To characterise focal adhesion dynamics, Schwarz and Erdmann had proposed a formulation where bond lifetime was dependent on cluster size, rebinding rate, and the force exerted at the adhesion [31]. In another study, Schwarz and co-workers used a two spring model to describe the contributions of cell-matrix adhesions to cellular activities, showing the influence of substrate stiffness on cell force built up [32]. Collectively, these studies indicate that in modelling cell-substrate interactions, cells and their underlying substrates are often modelled as elastic or viscoelastic solids, with simple description for capturing adhesion dynamics.

While de-adhesion experiments have been successful in demonstrating contractile modulation by ECM density, it remains unclear if the same assay can also be used for probing the cellular mechanoadaptation response on surfaces of varying ECM stiffness. In this paper, we present a computational framework for probing the collective influence of cell and substrate properties, distribution of adhesions and cell contractility in regulating cell-matrix de-adhesion dynamics. Here, the cell is modeled as a pre-stressed viscoelastic solid adhering to an elastic substrate via cell-matrix adhesions at a prescribed density. De-adhesion is simulated by rate-dependent chopping of bonds. By studying the dynamics of retraction, we demonstrate how the time scales are regulated by substrate properties and bond breakage rate, and determine analytical expressions of the time constants for certain regimes of the parameters. While comparing our model predictions with experimental de-adhesion curves of NIH 3T3 fibroblasts on polyacrylamide substrates of varying stiffness, we observe that contrary to delayed de-adhesion on stiffer substrates predicted by our model, fibroblasts exhibit faster de-adhesion on stiffer substrates. Finally, we show that this anomaly can be resolved by introducing a force-dependent bond breakage rate in our model. Taken together, our results demonstrate the utility of employing trypsin de-adhesion as a biophysical tool for probing mechanoadaptation and illustrate the complex dependence of de-adhesion time constants on substrate properties.

## Materials and Methods

### Fabrication of polyacrylamide hydrogels

Polyacrylamide hydrogels were fabricated as described elsewhere [33]. Briefly, glass coverslips were cleaned with 0.1 N NaOH, silanized with APTES for 5 mins (Sigma), rinsed with distilled water, and treated with 0.5% glutaraldehyde for 30 mins. Stocks of 40% acrylamide solutions and 2% bisacrylamide solutions (BioRad) were combined to obtain gels of 1.5 kPa, 2.5 kPa and 40 kPa stiffness. Finally, gels were covalently crosslinked with 10 *µ*g/ml rat tail collagen I (Sigma) using the photoactivable bi-functional crosslinker sulfo-SANPAH (Pierce).

### De-adhesion experiments

NIH 3T3 fibroblasts were maintained in T75 flasks in DMEM (Himedia) supplemented with 10% FBS (Invitrogen) at 37°C with 5% CO2. For experiments, fibroblasts were cultured on polyacrylamide hydrogels for a period of 24 hours. Quantification of cell spreading and cell shape was performed for at least 100 cells per condition across two independent experiments. For performing de-adhesion experiments, cells were washed with phosphate buffered saline (PBS, Himedia), incubated with 0.25% warm trypsin (Himedia) and immediately imaged in phase contrast using an inverted microscope (Olympus IX71) at 10× magnification. De-adhesion experiments were performed on a temperature controlled stage with temperature set at 37°C. Cells were imaged till they became rounded but remained attached to the substrate. Movies were processed manually in Image J (NIH) to obtain experimental de-adhesion curves (i.e., plots of normalized change in area versus time), which were fitted with Boltzmann curve to determine the de-adhesion time constants 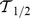 and 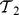. De-adhesion time constants were obtained for 15 cells per condition across two independent experiments.

### Computational Model

**Figure 1:**
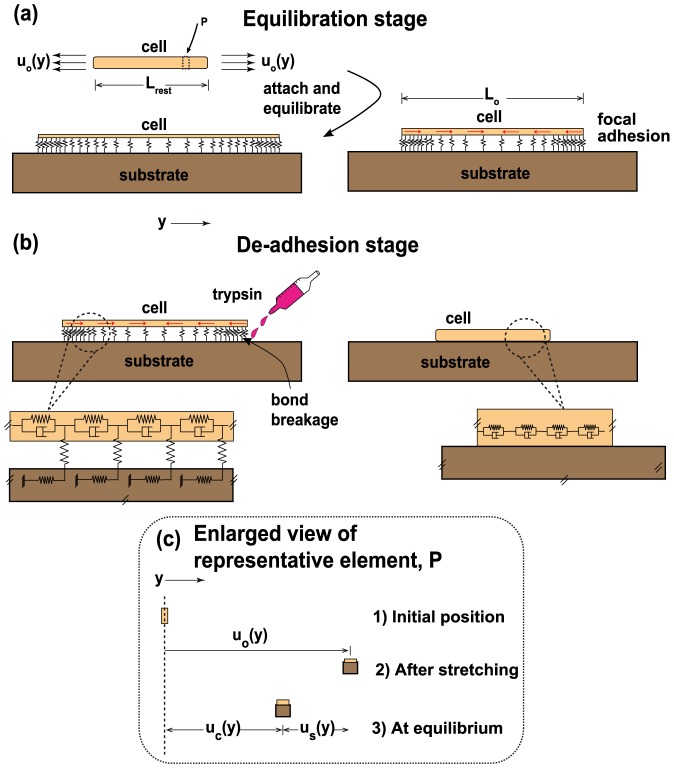
Schematic representation of the model. Cell is modeled as a viscoelastic solid (represented by springs and dashpots), and is physically coupled to the underlying elastic substrate through focal adhesions drawn as springs. The number of springs depend on the density of adhesions. (a) Contractility is imposed by stretching the cell and then coupling it to the underlying substrate through cell substrate bonds, till mechanical equilibrium is attained. Inward directed arrows inside the cell indicate the cell pre-stress. (b) De-adhesion was simulated by breaking bonds in a time-dependent manner till the cell reached the final rounded configuration (time 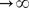) where all the adhesions were broken. The spatial positions of the cell in the fully stretched (a) and completely relaxed (b) states are shown. (c) Relationship between different displacement components of the system at equilibrium is shown using a representative element P.

To understand the role of substrate stiffness on experimentally observed de-adhesion pattern, we developed a one-dimensional (1–D) model, with the variables being stiffness of cell and substrate, cell contractility, number and distribution of cell-substrate bonds, and rate of bond breakage. Unlike complex cellular processes such as cell spreading or cell migration which occur in the timescale of tens of minutes to hours [1], [34], [35], and involve complex cytoskeletal reorganisation, de-adhesion involves rounding up of a 50–100 *µ*m size cell in a matter of seconds, where perhaps, cytoskeletal dynamics are less important. This has motivated us to have a simple viscoelastic description for the cell, as done in many other studies [26], [29], [30], [36]–[39]. Specifically, the cell was modeled as a 1–D viscoelastic object connected to the elastic substrate by a continuous distribution of bonds [32]. Various previous studies suggest that effects of cell contractility can be reasonably represented using pre-stress/pre-strain-based approaches [21], [40]–[42]. For the present study, we have adopted a similar method to incorporate cell contractility. In order to mimic contractility in our model, the cell, initially in a relaxed state, was brought to a stretched state by the application of a specified displacement field and attached to the substrate below with the help of cell-substrate bonds (see Fig. 1). Once the cell is connected to the substrate, it starts applying tractions on the substrate, and ultimately reaches mechanical equilibrium (tensional homeostasis) with the substrate resisting the internal pre-stress/contractile forces. Note that, in the absence of cell-substrate connections, no pre-stress buildup is possible.

For simulating adhesion and subsequent de-adhesion, the stretched configuration of the cell (at time 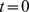) was generated by imposing an initial displacement field (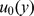) to a cell of rest length 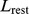 [21], and connecting it to the substrate through cell-substrate bonds (Fig. 1(a)). The equilibrated length of the cell is 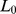. Thus, at time 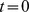, the cell is in a completely spread out state and in mechanical equilibrium with the substrate, the length co-ordinate being 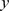. For this configuration, the stress along the cell can be written as 
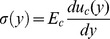
, where 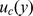 is the cell displacement at any point along the length of the cell. Due to the interaction of the cell with the substrate, the stress is also related to the substrate displacement, 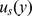. Thus, the force equilibrium equation along 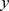 direction can be written as
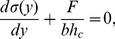
(1)where 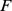 is the force transmitted (per unit length) by cell substrate bonds, 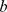 is the width and 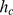 is the height of the cell. To get a relation between the substrate displacement 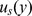 and traction, we assume that the force 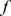, which is transmitted by a single cell-substrate bond, is distributed uniformly over a circle of size 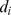, the diameter of the bond (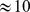
nm) [43], [44]. Further, assuming the substrate with Young's modulus 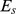 to be of much greater thickness as compared to the lateral extent of the cell, we use standard expressions to obtain a relation of the form 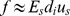, where 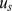 is the displacement of the center of the circle [45]. If the stiffness contributed from a single bond is 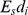, then the overall contribution to the substrate stiffness from 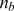 bonds is simply 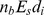. If 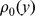 represents the density of adhesions per length along the length of the cell and 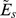 refers to the scaled effective stiffness of the substrate [43], [44] given by the expression 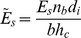, the force equilibrium in Eq. 1 can be written as
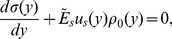
(2)


After writing the substrate displacement 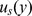 in terms of cell displacement as 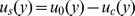, and non-dimensionalizing all length dimensions with 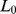, Eq. 2 becomes,
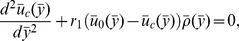
(3)where 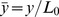, 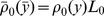 and the dimensionless parameter 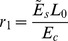. (In this paper, the convention followed for representing symbols is that a quantity with dimension of length or time is denoted with corresponding symbol and any parameter with a 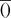 on top denote the corresponding dimensionless quantities of original equation). The displacement profile for the cell at 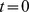 can be obtained by solving Eq. 3, with stress free boundary conditions, 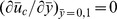. Previously, the bond density has been estimated to be of the order of 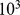 per square microns [46]. Since larger focal adhesions tend to be located at the cell periphery in many different cell types [47], significant rounding of the cell will occur only when these bonds are broken. Assuming a cell of 10 *µ*m radius and 1% adhesion belt at the periphery, one can estimate 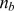 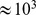. For cells cultured on ligand-coated glass coverslips, taking cell stiffness 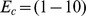 kPa and effective elasticity of ligand/glass complex to be 0.1 MPa [2], one can obtain estimates of 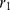 in the range of 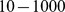.

De-adhesion was initiated by time-dependent irreversible breakage of bonds at a rate 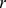 per unit time. Thus, upon chemical detachment of the bonds, the cell rounds up driven by the mechanical imbalance caused by the pre-stress in the cell. Finally, at time 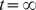, with all cell–substrate bonds broken, the length of the cell would be 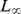 = 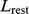 (fig. 1(b)). In our model, the stress along the cell at any time 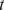 is written as:
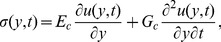
(4)where 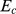 and 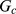 are the elastic and viscous modulus of the cell, respectively. As in the earlier case, the stress in cell is also related to the substrate displacement. The equilibrium equation can be written as
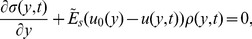
(5)


Taking the values of stress from Eq. 4, and re-writing substrate displacements in terms of cell displacements, Eq. 5 becomes as
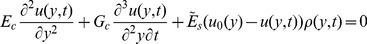
(6)


Non-dimensionalizing Eq. 6 using 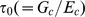 and 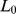 as metrics of time and length, respectively, we obtain the following non-dimensionalized version of Eq. 6:
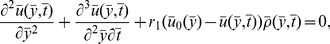
(7)where 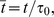 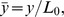 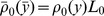 and 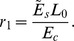 The displacements are naturally non-dimensionalized with 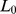. We have also assumed a simple rate dependence for bond breakage given by
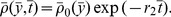
(8)


This is a standard procedure to describe first order rate kinetics of bond breaking or any similar kinetic events. It simply means that, we have used an equation of the following form 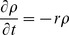.

The time-dependent cell displacement 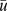 can be obtained by solving the above equation Eq. 7 using Eq. 8, subject to the following boundary conditions and initial condition:
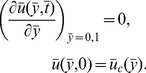
(9)


In all our simulations, instead of separately providing values of 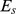, 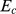, and other variables, we vary one of the three parameters mentioned in table 1.

**Table 1:** Description of parameters used in the study.

Variable	Description
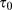	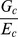
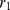	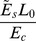
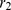	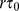

## Results

### De-adhesion of fibroblasts on hydrogels of varying stiffness

**Figure 2:**
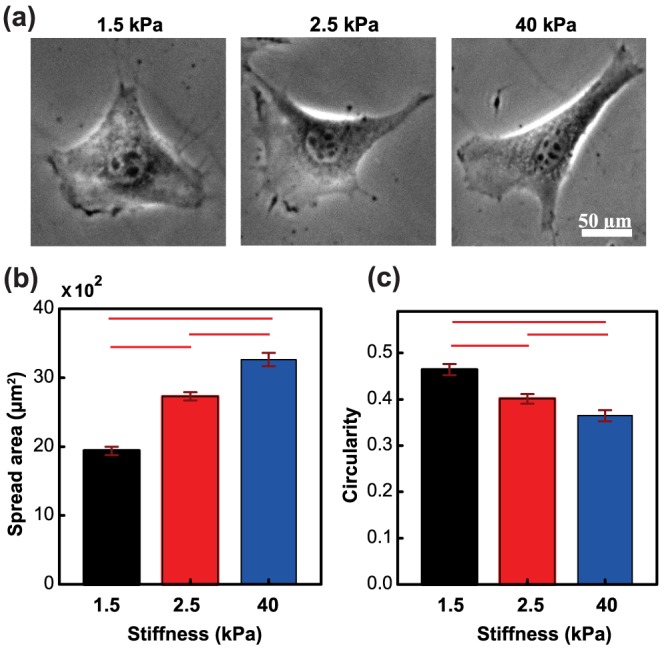
Quantification of cell spreading area and cell shape on substrates of varying stiffness. (a) Representative phase contrast images of NIH 3T3 fibroblasts cultured on 1.5, 2.5 and 40 kPa polyacrylamide gels for 24 hours. (b) Quantification of cell spreading area. Cell spreading increased with increasing substrate stiffness. Red bars denote statistical significance (*p<0.05). (c) Quantification of cell shape (circularity) on substrates of varying stiffness. Cell elongation increased with increasing substrate stiffness. Red bars denote statistical significance (*p<0.05).

**Figure 3:**
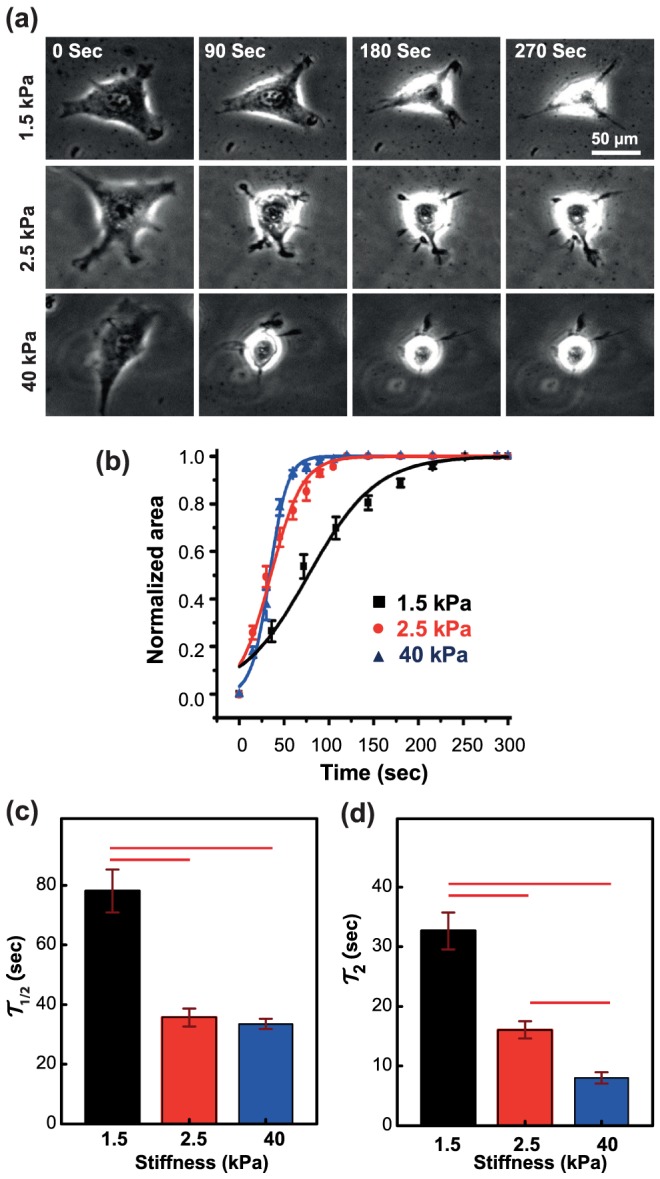
Trypsin induced de-adhesion of NIH 3T3 fibroblasts. NIH 3T3 fibroblasts were cultured on polyacrylamide hydrogels of stiffness 1.5, 2.5 and 40 kPa for 24 hours and then de-adhesion experiments were performed. (a) Upon treatment of trypsin, cells started rounding up. Sequence of time-lapse snaps of de-adhering cells on substrates of different stiffnesses are shown. (b) Plot of normalized area (defined as change in area at any time divided by net change in area during the entire de-adhesion process) as a function of time for cells de-adhering on different substrates. Fitting of experimental de-adhesion curves with Boltzman curve allowed us to determine the two time constants of de-adhesion– 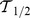 and 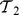, respectively. (c) and (d) show the plot of 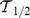 and 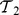 respectively, for de-adhering cells on three different substrate stiffnesses. Red bars denote statistical significance (*p<0.05).

Seminal work done by Janmey and co-workers demonstrated the mechanoadaptation response exhibited by fibroblasts cultured on substrates of varying stiffness [48]. Specifically, it was observed that fibroblasts match their cortical stiffness (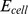) to that of substrate stiffness (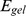) on substrates softer than 5 kPa, with cortical stiffness reaching saturation levels on substrates stiffer than 5 kPa. To probe how substrate stiffness influences de-adhesion timescales, NIH 3T3 fibroblasts were cultured on 3 different substrates (1.5 kPa, 2.5 kPa and 40 kPa) [33]. Two of these substrates were chosen softer than the 5 kPa threshold below which 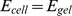 was observed by Solon et al. [48]; the third substrate was chosen significantly stiffer than 5 kPa. Consistent with previous cell-on-gel studies [8], [48], fibroblasts exhibited a stiffness-dependent spreading response with increased spreading on stiffer substrates (Fig. 2(b)). Cell shape was also found to change with cell stiffness, with cells exhibiting a more elongated morphology (i.e., low circularity) on stiffer substrates (Fig. 2(c)). While cells de-adhered slowest on the softest 1.5 kPa substrates, fastest de-adhesion was observed on the stiffest 40 kPa substrates (Fig. 3(a)). Fitting of experimental de-adhesion curves with Boltzman equation allowed us to quantitatively assess the influence of substrate stiffness on de-adhesion time constants (Fig. 3(b)). While 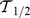 dropped from 80 sec observed on 1.5 kPa gels to 30 sec on 2.5 and 40 kPa gels, 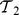 dropped from 30 sec observed on 1.5 kPa gels to 15 sec on 2.5 kPa gels and 8 sec on 40 kPa gels (Fig. 3(c, d)). Taken together, these results indicate that similar to cell stiffness, de-adhesion timescales are sensitive to substrate stiffness, and may be used as metrics for probing mechanoadaptation.

### Model predictions: Effect of substrate stiffness on de-adhesion timescales

**Figure 4:**
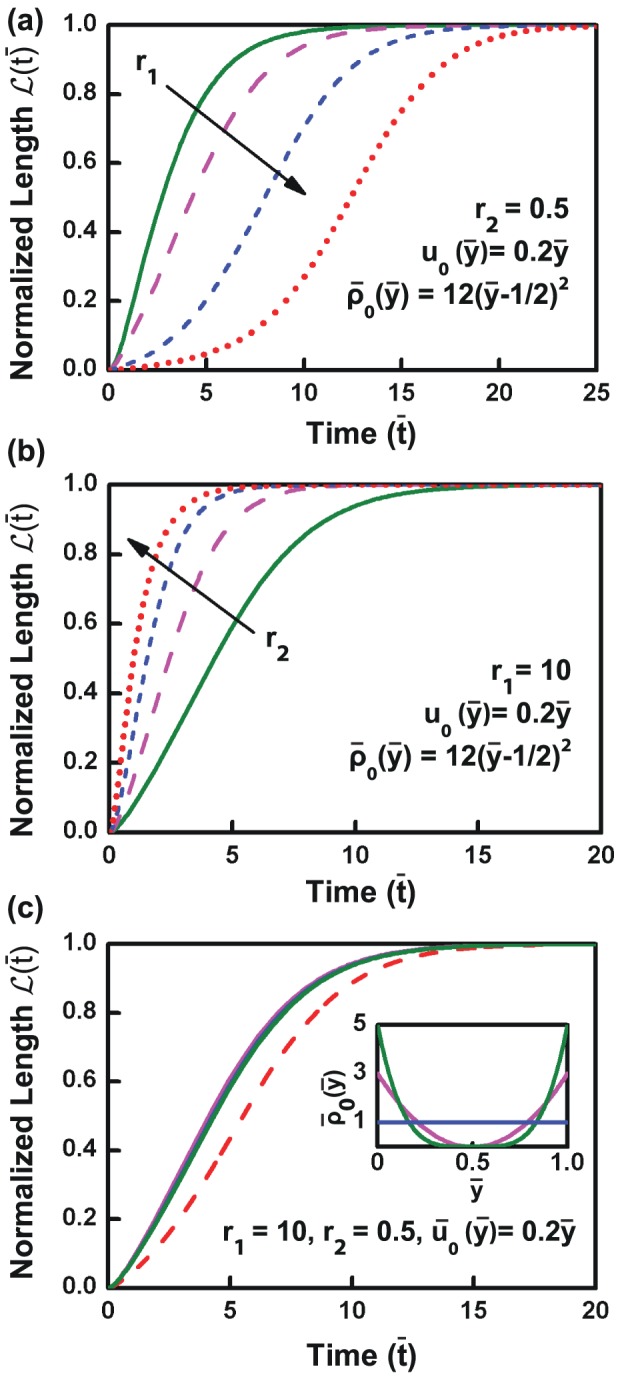
Influence of substrate properties, bond-breakage rate, and adhesion distribution on de-adhesion dynamics. (a) De-adhesion curves computed by varying stiffness ratio (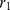) from 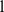 to 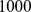 keeping 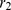, 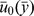 and 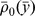 fixed (see text). (b) De-adhesion curves obtained for bond breakage rate (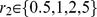) keeping 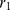, 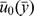, and 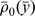 fixed. (c) Influence of distribution of adhesions on cellular retraction was assessed by comparing three different adhesion distributions (plotted in inset) while keeping all other variables unchanged. The dashed line corresponds to the case with adhesions at extreme ends of the cell, and gives the rightmost bound of the de-adhesion curves.

To quantitatively assess how different physical quantities influence de-adhesion dynamics, we tracked the length of the cell 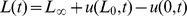 as a function of time. As is done in experiments [9], throughout the paper we present this length in a normalized fashion defined as 
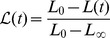
. To determine the influence of substrate stiffness on de-adhesion timescales, we varied the dimensionless parameter 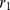, which represents the ratio of substrate stiffness to cell stiffness, and is referred to as stiffness ratio. To assess the sensitivity of stiffness ratio in regulating de-adhesion dynamics, we systematically varied 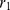 over three orders of magnitude from 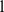 to 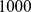, and tracked the normalized de-adhesion profiles (Fig. 4(a)). In doing these calculations, all other variables (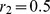, 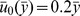 and 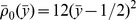) were kept unchanged. Under these conditions, fastest de-adhesion was observed for 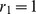 with a half saturation time constant 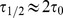 and slowest for 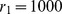 with a half saturation time constant 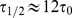 where 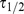 is the time when 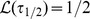. Though these results illustrate the influence of stiffness ratio on de-adhesion timescales, these predictions contradict our experimental findings, wherein faster de-adhesion was observed on stiffer substrates.

### Model predictions: Influence of adhesion strength and adhesion distribution on de-adhesion timescales

De-adhesion is expected to be influenced by the strength of bonds formed between cells and their substrate. In addition, several experimental studies have highlighted the effect of substrate stiffness in influencing the formation and dynamics of focal adhesions [49], [50]. To directly determine the influence of adhesion strength on de-adhesion profiles, we computed the de-adhesion profiles for different values of the non-dimensional bond cutting rate 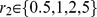 while keeping all the other variables fixed. This rate is representative of the characteristic time required for breakage of adhesions, and, is therefore, indicative of adhesion strength of the bonds. As seen in Fig. 4(b), 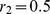 led to a de-adhesion profile with a half saturation 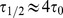. A ten-fold increase in 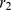 led to a significantly faster de-adhesion response with 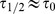. Subsequent increase in 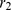 led to faster de-adhesion with saturation for 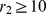. Moreover, the shape of the de-adhesion profile changed from a sigmoidal curve to that of a single exponential 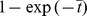 for high values of 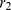 reflecting the internal relaxation time 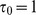 of the cell.

Though these calculations demonstrate the influence of bond breakage rate in setting de-adhesion time scales, in all the above calculations, the distribution of adhesions 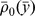 remained the same. However experiments with a range of different cell types have demonstrated that cells form bigger adhesions in peripheral regions, and smaller sized adhesions towards the centre. To study the influence of distribution of adhesions, we compared the de-adhesion profiles arising from three different patterns of adhesion 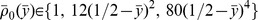 while keeping all the other variables constant (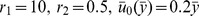). Further, the distributions of adhesions were chosen in a manner so as to ensure that the total number of adhesions remained constant. The resulting de-adhesion plots are shown in Fig. 4(c). In comparison to the first case where adhesion was uniform across the entire length of the cell, the other two distributions were non-uniform but symmetric about the cell centre with strongest adhesions at the two ends and no adhesion in the centre. As seen in Fig. 4(c), these two distributions yielded nearly the same de-adhesion profile suggesting that de-adhesion profile is independent of the adhesion distribution. An extreme limit of bond-distribution is when 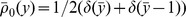, where 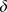 is the Dirac-delta function; this is when the adhesions are concentrated only on the two extreme edges. In such a limit, we need to track only the edges of the cell, and the governing equation of motion will be reduced to an ordinary differential equation:
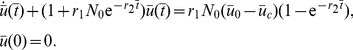
(10)


Here, 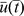 is the cell displacement and 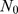 is the total number of bonds at equilibrium, respectively, at the end of the cell. All other symbols have their usual meaning. It can be seen from the dotted line in Fig. 4(c) that this limit places a right bound on the de-adhesion profiles.

### Analytical estimates of 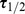 and 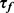


**Figure 5:**
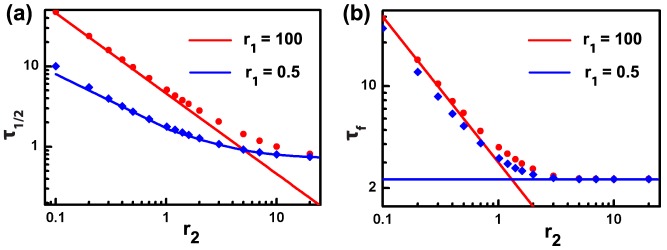
Analytical estimates of de-adhesion time scales. 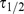
 and 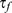 are plotted as functions of 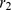 for two values of 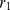: 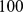 (red points) and 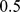 (blue points). The corresponding colour lines are fits to various analytical expressions described in the text.

Thus far, our simulations have allowed us to asses the individual influence of stiffness ratio (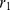) and bond-breaking rate (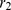) on the de-adhesion response. Now we study their collective influence and identify different regimes where each of these parameters play a dominant role. From the computed de-adhesion profiles, two time constants –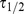 and 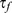
–were extracted, so that we could quantitatively study their dependence on 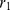 and 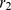. While 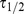 is the time needed to reach 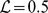, 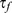 is the time required to reach 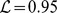 from 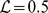. Even though we see in previous section that de-adhesion time scales are sensitive to both 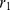 and 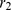, the extend to which 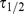 and 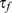 depend on 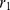 and 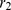 remains unclear. To get some insights, 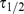 and 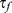 were plotted for increasing values of 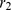 for 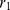 = 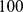 (red points) and 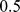 (blue points) (Figs. 5(a) and (b)). Irrespective of the choice of 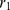, increase in 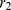 led to faster de-adhesion with fastest de-adhesion time scales of 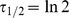 and 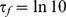, respectively. Under certain limiting conditions for the parameters 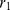 and 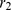, we were able to obtain approximate closed form expressions for 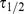 and 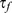. They are plotted as lines in the respective plots to demonstrate their accuracy of fitting. In the regime 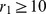 and 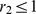, the expression 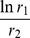 (red line, Fig. 5(a)) is seen to be a very good estimate of 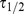. The corresponding expression for 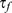 is given by 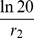 and works well for 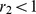 (red line, Fig. 5(b)). For very low values of 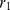, 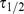 is given by solution of the equation 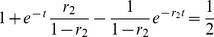 (Fig. 5(a), blue line) and follows the computed solution very closely. For large 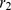, 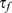 converges to 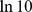 irrespective of 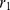 (Fig. 5(b), blue line).

### Model predictions: Influence of force dependent bond breakage on de-adhesion timescales

Till now, we have addressed the influence of substrate properties and cell-substrate adhesions in regulating de-adhesion dynamics and shown that stiffness ratio and bond-breakage rate play important roles in setting de-adhesion time scales. Further, the analytical insights obtained from the model provide a quantitative understanding of how various physical parameters govern the de-adhesion profile. Though insightful, this model, however, failed to reproduce the experimental observations, perhaps due to its insensitivity to the magnitude of contractility (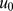), i.e., for any non-zero magnitude of contractility this model produces identical de-adhesion curves. This insensitivity is due to the linearity of the model in 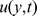 because of which, the magnitude 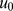 of contractility gets cancelled out from the numerator and denominator in the expression for normalised length 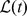. As explained below, we address this shortcoming in the current model by coupling contractility and bond-breakage.

**Figure 6:**
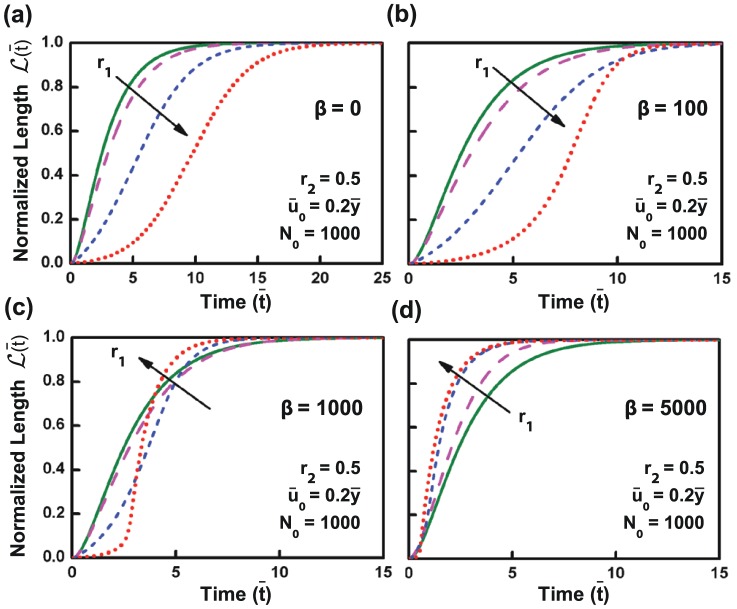
Influence of force-dependent bond breakage on de-adhesion dynamics. Force-dependent bond breakage was simulated by varying the parameter, 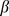. Figure (a) - (d) show the de-adhesion profile for different values of 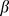 for varying stiffness ratio (0.1–100). While delayed de-adhesion on stiffer substrates was observed for low values of 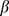 (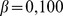), faster de-adhesion was observed on stiffer substrates at higher values of 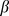 (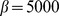).

Several theoretical and experimental studies in the past have demonstrated that bond lifetimes are sensitive to forces [51]–[54]. To capture the effect of force dependence, it was assumed that bond breakage happens in a force dependent manner [55]–[57] given by the expression
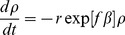
(11)where 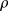 is the bond density as in previous sections, and 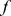 represents the force acting on the bonds. All other variables have the same meaning as earlier. In order to understand the de-adhesion dynamics of cell for this force-dependent bond breakage conditions, simulations were done as earlier and corresponding de-adhesion profiles obtained for varying values of 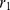 (0.1–100) (Fig. 6). Interestingly, 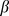 is seen to play an important role in determining the de-adhesion profiles. As the value of constant 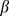 increases, the nature of de-adhesion profile also reverses. Figure 6 (a)–(d) show that for lower values of 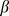, de-adhesion happens faster on softer substrates. In contrast, when 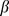 is very high, the nature of de-adhesion profile changes and fastest de-adhesion occurs for highest stiffness, which matches with the experimental results (Fig. 3).

### Model predictions: Influence of contractility on de-adhesion timescales

**Figure 7:**
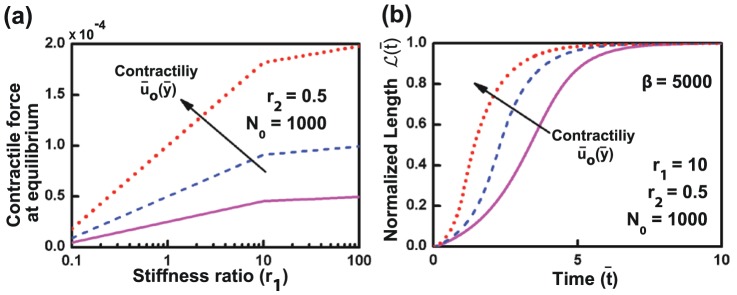
Influence of cell contractility on de-adhesion dynamics. The effect of cell contractility on de-adhesion dynamics was studied for three contractility values: 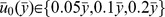 at the cell end, while keeping other parameters fixed. (a) Plot of contractile force at equilibrium for varying stiffness ratios for different contractility values. (b) Influence of cell contractility on de-adhesion dynamics, with other parameters kept constant. On a substrate of given stiffness, higher contractility led to faster de-adhesion.

In our mathematical model, contractility is introduced by applying a pre-stretch to the initially relaxed cell and then attaching the cell to the substrate below by cell-substrate bonds. This way, the cell is brought to a pre-stressed state before the beginning of de-adhesion process itself, with the applied displacement field, 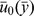 being a measure of cell contractility, i.e., higher the value of 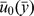, greater will be the cell pre-stress. Thus, the effect of substrate stiffness on cell contractility is implicit in our model. The exact nature of this dependence can be seen by plotting the contractile force at equilibrium for different values of 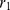 and varying 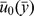. As seen in Figure 7(a), it is observed that for a given initial displacement field (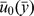), the pre-stress (at 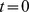) initially increases with substrate stiffness and then saturates. Moreover, higher contractility on higher stiffness substrates, or, higher contractility on identical stiffness substrates (effected experimentally by contractility-activating drugs), would predict faster de-adhesion. This is clearly seen in de-adhesion profiles plotted for three different contractility values (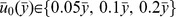) (Fig. 7(b)), where higher contractility drives faster de-adhesion.

## Discussion

In this paper, we have combined experimental and computational approaches to understand how substrate stiffness influences trypsin-induced de-adhesion of fibroblasts. First, by doing de-adhesion experiments on substrates of varying stiffness, we have shown that cells on stiffer substrates de-adhere faster, driven by higher baseline contractility, with blebbistatin treatment nullifying the stiffness-dependent effects. To directly probe the contributions of various factors to de-adhesion, a computational model was developed incorporating the effects of stiffness ratio, strength and distribution of adhesions, and the contractility. Analytical estimates obtained for certain regimes of stiffness ratio and bond breakage rate demonstrate the coupled dependence of de-adhesion timescales on the two parameters. Comparison of the computed stiffness-dependent de-adhesion profiles with the experimental de-adhesion curves suggest that faster de-adhesion on stiffer substrates is predicted by a force-dependent bond breakage rate. Collectively, our results highlight the direct influence of substrate stiffness in regulating de-adhesion time scales.

Cell spreading, proliferation, differentiation and invasion have all been shown to depend on substrate stiffness, and are intimately tied to the mechanical state of the cell. Specifically, substrate stiffness has been shown to modulate the cortical stiffness of NIH 3T3 fibroblasts in a profound manner, with cortical stiffness closely matching the substrate stiffness upto 5 kPa, and reaching a saturation value on stiffer substrates [48]. Similar mechanoadaptation responses has also been documented in cancer cells, and require both an intact actin cytoskeleton and focal adhesion proteins [8], [58]. Since higher myosin II activity increases traction forces [59], [60] and cortical stiffness measurements [8], [61], we reasoned that trypsin-induced de-adhesion which is driven by cell contractility [9], [10], can be used as an assay for probing cell mechanoadaptation. Consistent with our hypothesis, we show here that cells de-adhere faster in comparison to cells on softer substrates. The ease of conducting de-adhesion experiments, the simplicity of analysis makes it ideally suited for probing contractile mechanics and cellular mechanoadaptation responses in adherent cells. However, it remains to be seen if similar substrate stiffness-dependent de-adhesion responses are exhibited by other cell types.

De-adhesion-induced rounding requires simultaneous breakage of cell-matrix adhesions and cell retraction. Consequently, de-adhesion timescales depend both on the initial state of the cystokeleton and the rate of bond breakage. This has been demonstrated in previous experiments where contractile stimulation via drugs including LPA and nocodazole, or faster dis-assembly of adhesions effected by using a higher concentration of trypsin, both led to faster de-adhesion [9], [10]. Such a coupled dependency of 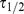 on the two factors can be appreciated from the analytical expression 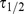 = 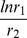. For a constant bond cutting rate (i.e., constant 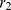), this equation predicts delayed de-adhesion of cells on stiffer substrates (i.e., increase in the value of 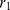). Though this prediction directly contradicts our experimental observations where cells on stiffer substrates de-adhere faster compared to those on softer substrates, the utility of the analytical expressions of de-adhesion timescales obtained for force-independent bond breakage lie in demonstrating the dependence of de-adhesion timescales not on stiffness directly but on logarithm of stiffness. This is why increase of stiffness from 1.5 kPa to 40 kPa reduces 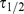 by 50% only. At high bond cutting rates, the de-adhesion timescales are relatively insensitive to substrate properties. Together, these results suggest that the combination of substrate properties and bond severing rates determine de-adhesion timescales.

Several experimental studies have demonstrated that both cell spreading and traction forces increase with substrate stiffness. This is made possible by the formation of larger adhesions, which are also more stable and experience larger forces. Additionally, experimental and theoretical studies on dissociation of bonds under force have demonstrated that dissociation rate of a bond increases exponentially under the presence of a force. This would suggest that in de-adhesion experiments, disruption of a single focal adhesion leads to enhanced load on the other bonds, thereby reducing their bond lifetimes. Force-dependent bond breakage has been incorporated in our simulations through the parameter 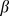. Similar to the force-independent bond breakage results, delayed de-adhesion on stiffer substrates was observed for low values of 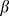 ( = 100 and less). This trend started to get reversed for values of 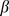 in the range of 1000, and, for high values of 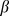 ( = 5000), similar to experimental observations, faster de-adhesion was observed on stiffer substrates. The simulation results thus suggest that the nature of stiffness-dependent de-adhesion response depends on the magnitude of contractile forces exerted by cells. It is conceivable that a delayed stiffness-dependent de-adhesion response may be observed in cells which are less spread and possess low baseline contractility.

As is widely known, intracellular contractility is one of the key parameters which influences process of adhesion, as well as, the process of de-adhesion as studied here. Motivated by similar studies in the past [21], [40], [41], we have adopted a pre-stress-based approach to model cell contractility. In conjunction with substrate stiffness, the initial displacement field 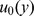 sets the magnitude of cell contractility, thereby accounting for the effect of substrate stiffness on cell contractility (Fig. 7). Consistent with previous studies [62], our model suggests a direct dependence of cell pre-stress on substrate stiffness. Specifically, for a given pre-stretch, with increase in substrate stiffness, pre-stress initially increases and then saturates on stiffer substrates. Furthermore, on a substrate of given stiffness, as contractility increases, cell de-adhere faster, as is experimentally observed in faster de-adhesion of cells treated with contractility-activating drugs like LPA and nocodazole [9], [10]. Collectively, these results demonstrate how substrate stiffness influences de-adhesion dynamics via modulation of cell contractility.

In conclusion, in this study, we have addressed the influence of substrate properties and cell-substrate adhesions in regulating de-adhesion dynamics. We have shown that de-adhesion timescales can be used as metrics for quantifying mechanoadaptation responses, and that both stiffness ratio and bond-breakage rate play important roles in setting de-adhesion time scales.

## References

[1] Doyle AD, Wang FW, Matsumoto K, Yamada KM (2009) One-dimensional topography underlies three-dimensional fibrillar cell migration. J Cell Biol 184: 481–490. 19221195 10.1083/jcb.200810041 PMC2654121

[2] Engler A, Bacakova L, Newman C, Hategan A, Griffin M, et al (2004) Substrate compliance versus ligand density in cell on gel responses. Biophys J 86: 617–628. 14695306 10.1016/S0006-3495(04)74140-5 PMC1303831

[3] Ulrich TA, de Juan Pardo EM, Kumar S (2009) The mechanical rigidity of the extracellular matrix regulates the structure, motility, and proliferation of glioma cells. Cancer Res 69: 4167–4174. 19435897 10.1158/0008-5472.CAN-08-4859 PMC2727355

[4] Engler AJ, Griffin MA, Sen S, Bonnemann CG, Sweeney HL, et al (2004) Myotubes differentiate optimally on substrates with tissue-like stiffness pathological implications for soft or stiff microenvironments. J Cell Biol 166: 877–887. 15364962 10.1083/jcb.200405004 PMC2172122

[5] Levental KR, Yu H, Kass L, Lakins JN, Egeblad M, et al (2009) Matrix crosslinking forces tumor progression by enhancing integrin signaling. Cell 139: 891–906. 19931152 10.1016/j.cell.2009.10.027 PMC2788004

[6] Discher DE, Janmey P, Wang Yl (2005) Tissue cells feel and respond to the stiffness of their substrate. Science 310: 1139–1143. 16293750 10.1126/science.1116995

[7] Safran S, Gov N, Nicolas A, Schwarz U, Tlusty T (2005) Physics of cell elasticity, shape and adhesion. Phys A Stat Mech its Appl 352: 171–201.

[8] Sen S, Dong M, Kumar S (2009) Isoform-specific contributions of *α*-actinin to glioma cell mechanobiology. PLoS One 4: e8427. 20037648 10.1371/journal.pone.0008427 PMC2793025

[9] Sen S, Kumar S (2009) Cell–matrix de-adhesion dynamics reflect contractile mechanics. Cell Mol Bioeng 2: 218–230. 21297858 10.1007/s12195-009-0057-7 PMC3018270

[10] Sen S, Ng WP, Kumar S (2011) Contractility dominates adhesive ligand density in regulating cellular de-adhesion and retraction kinetics. Ann Biomed Eng 39: 1163–1173. 21046466 10.1007/s10439-010-0195-z PMC3069333

[11] Kapoor A, Sen S (2012) Synergistic modulation of cellular contractility by mixed extracellular matrices. Int J Cell Bio 471591. 10.1155/2012/471591 PMC3517853 23251159

[12] Das A, Kapoor A, Mehta G, Ghosh S, Sen S (2013) Extracellular matrix density regulates extracellular proteolysis via modulation of cell contractility. J Carcinogenesis and Mutagenesis S13.

[13] Soumya SS, Kolwankar S, George E, Basu SK, Sen S, et al (2014) Spatial anisotropy and heterogeneity in contractility and adhesion distribution may contribute to cell steering during migration. Appl Phys Lett 104: 083705.

[14] Harland B, Walcott S, Sun SX (2011) Adhesion dynamics and durotaxis in migrating cells. Phys Biol 8: 015011. 21301061 10.1088/1478-3975/8/1/015011 PMC4479195

[15] Ronan W, Deshpande VS, McMeeking RM, McGarry JP (2014) Cellular contractility and substrate elasticity: a numerical investigation of the actin cytoskeleton and cell adhesion. Biomech Model Mechanobiol 13: 417–435. 23775256 10.1007/s10237-013-0506-z

[16] Dowling EP, McGarry JP (2014) Influence of spreading and contractility on cell detachment. Ann Biomed Eng 42: 1037–1048. 24356853 10.1007/s10439-013-0965-5

[17] Zeng X, Li S (2014) Biomechanical cell model by liquid-crystal elastomers. J Eng Mech 140: 04013003 1–10.

[18] Friedrich BM, Safran SA (2012) How cells feel their substrate: spontaneous symmetry breaking of active surface stresses. Soft Matter 8: 3223–3230.

[19] Lin YC, Tambe DT, Park CY, Wasserman MR, Trepat X, et al (2010) Mechanosensing of substrate thickness. Phys Rev E 82: 041918. 10.1103/PhysRevE.82.041918 PMC3641827 21230324

[20] Zaman MH, Kamm RD, Matsudaira P, Lauffenburger DA (2005) Computational model for cell migration in three-dimensional matrices. Biophys J 89: 1389–1397. 15908579 10.1529/biophysj.105.060723 PMC1366623

[21] Edwards CM, Schwarz US (2011) Force localization in contracting cell layers. Phys Rev Lett 107: 128101. 22026803 10.1103/PhysRevLett.107.128101

[22] Zemel A, Rehfeldt F, Brown A, Discher D, Safran S (2010) Optimal matrix rigidity for stress-fibre polarization in stem cells. Nat Phys 6: 468–473. 20563235 10.1038/nphys1613 PMC2885792

[23] Banerjee S, Marchetti MC (2012) Contractile stresses in cohesive cell layers on finite-thickness substrates. Phys Rev Lett 109: 108101. 23005331 10.1103/PhysRevLett.109.108101

[24] Banerjee S, Marchetti MC (2013) Controlling cell–matrix traction forces by extracellular geometry. New J Phys 15: 035015.

[25] Rebelo L, de Sousa J, Mendes Filho J, Radmacher M (2013) Comparison of the viscoelastic properties of cells from different kidney cancer phenotypes measured with atomic force microscopy. Nanotechnology 24: 055102. 23324556 10.1088/0957-4484/24/5/055102

[26] Coskun H, Li Y, Mackey MA (2007) Ameboid cell motility: A model and inverse problem, with an application to live cell imaging data. J Theor Biol 244: 169–179. 16997326 10.1016/j.jtbi.2006.07.025

[27] Park S, Koch D, Cardenas R, Käs J, Shih C (2005) Cell motility and local viscoelasticity of fibroblasts. Biophys J 89: 4330–4342. 16199496 10.1529/biophysj.104.053462 PMC1366997

[28] Yamada S, Wirtz D, Kuo SC (2000) Mechanics of living cells measured by laser tracking microrheology. Biophys J 78: 1736–1747. 10733956 10.1016/S0006-3495(00)76725-7 PMC1300770

[29] Yoon SH, Lee C, Mofrad MR (2011) Viscoelastic characterization of the retracting cytoskeleton using subcellular detachment. Appl Phys Lett 98: 133701.

[30] Yoon SH, Mofrad MR (2011) Cell adhesion and detachment on gold surfaces modified with a thiol-functionalized rgd peptide. Biomaterials 32: 7286–7296. 21813174 10.1016/j.biomaterials.2011.05.077

[31] Erdmann T, Schwarz U (2004) Stability of adhesion clusters under constant force. Phys Rev Lett 92: 108102. 15089248 10.1103/PhysRevLett.92.108102

[32] Schwarz US, Erdmann T, Bischofs IB (2006) Focal adhesions as mechanosensors: the two-spring model. Biosystems 83: 225–232. 16236431 10.1016/j.biosystems.2005.05.019

[33] Tse JR, Engler AJ (2010) Preparation of hydrogel substrates with tunable mechanical properties. Curr Protoc Cell Biol 10: 10–16. 10.1002/0471143030.cb1016s47 20521229

[34] Reinhart-king CA, Dembo M, Hammer DA (2005) The Dynamics and Mechanics of Endothelial Cell Spreading. Biophys J 89: 676–689. 15849250 10.1529/biophysj.104.054320 PMC1366566

[35] Cavalcanti-Adam EA, Volberg T, Micoulet A, Kessler H, Geiger B, et al (2007) Cell spreading and focal adhesion dynamics are regulated by spacing of integrin ligands. Biophys J 92: 2964–74. 17277192 10.1529/biophysj.106.089730 PMC1831685

[36] Mesquita A, Coda H (2003) A simple kelvin and boltzmann viscoelastic analysis of three-dimensional solids by the boundary element method. Eng Anal Bond Elem 27: 885–895.

[37] Karcher H, Lammerding J, Huang H, Lee RT, Kamm RD, et al (2003) A three-dimensional viscoelastic model for cell deformation with experimental verification. Biophys J 85: 3336–3349. 14581235 10.1016/S0006-3495(03)74753-5 PMC1303611

[38] Jamali Y, Azimi M, Mofrad MR (2010) A sub-cellular viscoelastic model for cell population mechanics. PloS One 5: e12097. 20856895 10.1371/journal.pone.0012097 PMC2938372

[39] Larripa K, Mogilner A (2006) Transport of a 1d viscoelastic actin–myosin strip of gel as a model of a crawling cell. Phys A Stat Mech its Appl 372: 113–123. 10.1016/j.physa.2006.05.008 PMC2600887 19079754

[40] He S, Su Y, Ji B, Gao H (2014) Some basic questions on mechanosensing in cell-substrate interaction. J Mech Phys Solids 70: 116–135.

[41] Sen S, Engler AJ, Discher DE (2009) Matrix strains induced by cells: computing how far cells can feel. Cell Mol Bioeng 2: 39–48. 20582230 10.1007/s12195-009-0052-z PMC2891090

[42] Schwarz US, Safran SA (2013) Physics of adherent cells. Rev Mod Phys 85: 1327–1381.

[43] Sens P (2013) Rigidity sensing by stochastic sliding friction. Euro Phys Lett 104: 38003.

[44] Walcott S, Sun SX (2010) A mechanical model of actin stress fiber formation and substrate elasticity sensing in adherent cells. Proc Natl Acad Sci USA 107: 7757–7762. 20385838 10.1073/pnas.0912739107 PMC2867880

[45] Landau L, Lifshitz EM (2005) Theory of elasticity. UK: Butterworth–Heinemann.

[46] Oberhauser AF, Badilla-Fernandez C, Carrion-Vazquez M, Fernandez JM (2002) The mechanical hierarchies of fibronectin observed with single-molecule afm. J Mol Biol 319: 433–447. 12051919 10.1016/S0022-2836(02)00306-6

[47] Balaban NQ, Schwarz US, Riveline D, Goichberg P, Tzur G, et al (2001) Force and focal adhesion assembly: a close relationship studied using elastic micropatterned substrates. Nat Cell Biol 3: 466–472. 11331874 10.1038/35074532

[48] Solon J, Levental I, Sengupta K, Georges PC, Janmey PA (2007) Fibroblast adaptation and stiffness matching to soft elastic substrates. Biophys J 93: 4453–4461. 18045965 10.1529/biophysj.106.101386 PMC2098710

[49] Pelham RJ, Wang YL (1997) Cell locomotion and focal adhesions are regulated by substrate flexibility. Proc Natl Acad Sci (USA) 94: 13661–13665. 9391082 10.1073/pnas.94.25.13661 PMC28362

[50] Prager-Khoutorsky M, Lichtenstein A, Krishnan R, Rajendran K, Mayo A, et al (2011) Fibroblast polarization is a matrix-rigidity-dependent process controlled by focal adhesion mechanosensing. Nat Cell Biol 13: 1457–1465. 22081092 10.1038/ncb2370

[51] Bell GI, Dembo M, Bongrand P (1984) Cell adhesion. competition between nonspecific repulsion and specific bonding. Biophys J 45: 1051–1064. 6743742 10.1016/S0006-3495(84)84252-6 PMC1434996

[52] Hammer DA, Lauffenburger DA (1987) A dynamical model for receptor-mediated cell adhesion to surfaces. Biophys J 52: 475–487. 2820521 10.1016/S0006-3495(87)83236-8 PMC1330012

[53] Evans E, Ritchie K (1997) Dynamic strength of molecular adhesion bonds. Biophys J 72: 1541–1555. 9083660 10.1016/S0006-3495(97)78802-7 PMC1184350

[54] Evans EA, Calderwood DA (2007) Forces and bond dynamics in cell adhesion. Science 316: 1148–1153. 17525329 10.1126/science.1137592

[55] Bell GI (1978) Models for the specific adhesion of cells to cells. Science 200: 618–627. 347575 10.1126/science.347575

[56] Gao H, Qian J, Chen B (2011) Probing mechanical principles of focal contacts in cell-matrix adhesion with a coupled stochastic-elastic modelling framework. J Royal Soc Int 8: 1217–32. 10.1098/rsif.2011.0157 PMC3140725 21632610

[57] Isabey D, Fereol S, Caluch A, Fodil R, Louis B, et al (2013) Force distribution on multiple bonds controls the kinetics of adhesion in stretched cells. J Biomech 46: 307–313. 23178039 10.1016/j.jbiomech.2012.10.039

[58] Sen S, Ng WP, Kumar S (2012) Contributions of talin-1 to glioma cell-matrix tensional homeostasis. J Royal Soc Int 9: 1311–1317. 10.1098/rsif.2011.0567 PMC3350720 22158841

[59] Lo CM, Wang HB, Dembo M, Wang YL (2000) Cell movement is guided by the rigidity of the substrate. Biophys J 79: 144–152. 10866943 10.1016/S0006-3495(00)76279-5 PMC1300921

[60] Kong HJ, Polte TR, Alsberg E, Mooney DJ (2005) Fret measurements of cell-traction forces and nano-scale clustering of adhesion ligands varied by substrate stiffness. Proc Natl Acad Sc USA 102: 4300–4305. 15767572 10.1073/pnas.0405873102 PMC555469

[61] Sen S, Tewari M, Zajac A, Barton E, Sweeney HL, et al (2011) Upregulation of paxillin and focal adhesion signaling follows dystroglycan complex deletions and promotes a hypertensive state of differentiation. Eur J Cell Biol 90: 249–260. 20663583 10.1016/j.ejcb.2010.06.005 PMC2970638

[62] Engler AJ, Sen S, Sweeney HL, Discher DE (2006) Matrix elasticity directs stem cell lineage specification. Cell 126: 677–689. 16923388 10.1016/j.cell.2006.06.044

